# Development of novel detection system for sweet potato leaf curl virus using recombinant scFv

**DOI:** 10.1038/s41598-020-64996-0

**Published:** 2020-05-15

**Authors:** Sang-Ho Cho, Eui-Joon Kil, Sungrae Cho, Hee-Seong Byun, Eun-Ha Kang, Hong-Soo Choi, Mi-Gi Lee, Jong Suk Lee, Young-Gyu Lee, Sukchan Lee

**Affiliations:** 1 College of Biotechnology and Bioengineering, Sungkyunkwan University, Suwon, 16419 Republic of Korea; 2 Department of Plant Medicals, Andong National University, Andong, 36729 Republic of Korea; 3 Crop Protection Division, National Academy of Agricultural Science, Rural Development Administration, Wanju, 55365 Republic of Korea; 4 Bio-Center, Gyeonggido Business and Science Accelerator, Suwon, 16229 Republic of Korea; 5 Highland Agriculture Research Institute, National Institute of Crop Science, Rural Development Administration, Pyeongchang, 25342 Republic of Korea

**Keywords:** Phage biology, Microbe

## Abstract

Sweet potato leaf curl virus (SPLCV) causes yield losses in sweet potato cultivation. Diagnostic techniques such as serological detection have been developed because these plant viruses are difficult to treat. Serological assays have been used extensively with recombinant antibodies such as whole immunoglobulin or single-chain variable fragments (scFv). An scFv consists of variable heavy (V_H_) and variable light (V_L_) chains joined with a short, flexible peptide linker. An scFv can serve as a diagnostic application using various combinations of variable chains. Two SPLCV-specific scFv clones, F7 and G7, were screened by bio-panning process with a yeast cell which expressed coat protein (CP) of SPLCV. The scFv genes were subcloned and expressed in *Escherichia coli*. The binding affinity and characteristics of the expressed proteins were confirmed by enzyme-linked immunosorbent assay using SPLCV-infected plant leaves. Virus-specific scFv selection by a combination of yeast-surface display and scFv-phage display can be applied to detection of any virus.

## Introduction

The sweet potato (*Ipomoea batatas* L.) ranks among the world’s seven most important food crops, along with wheat, rice, maize, potato, barley, and cassava^1,2^. Because sweet potatoes propagate vegetatively, rather than through seeds, they are vulnerable to many diseases, including viruses^3^. Once infected with a virus, successive vegetative propagation can increase the intensity and incidence of a disease, resulting in uneconomical yields.

**Figure 1:**
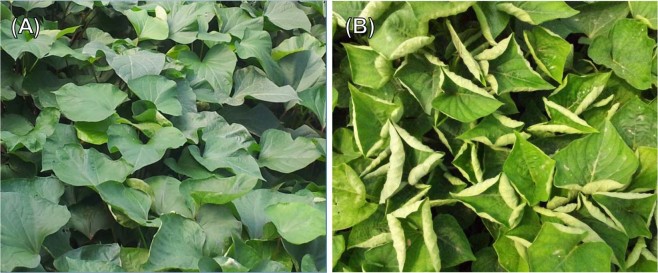
(**A**) Healthy sweet potato leaves. **(B)** Symptomatic leaves from sweet potato leaf curl virus-infected sweet potato plants.

Geminiviruses have a twin icosahedral-particle morphology and their DNA consists of circular single-stranded genomes of approximately 3.0 kb^4–6^. Geminiviruses are classified into four genera, *Mastrevirus, Begomovirus, Curtovirus* and *Topocuvirus*, based on their insect vector^7^. The sweet potato leaf curl virus (SPLCV), a member of the genus *Begomovirus*, is transmitted by the whitefly (*Bemisia tabaci* Genn.), which is the only natural vector^8^. SPLCV, which causes symptoms including upward leaf curling in young stage (Fig. 1B), is responsible for declining yields around the world^9,10^.

Efficient diagnosis and rapid treatment are important elements of any disease-control strategy. Several detection methods employing polymerase chain reaction (PCR) of SPLCV genomic DNA have been reported^11–13^. PCR is used widely to detect plant viruses in infected tissues^14–16^. However, PCR suffers from several disadvantages, such as the requirement for complete nucleic acid isolation and costly diagnostic equipment such as a thermocycler and UV transilluminator.

Serological diagnostic techniques are often preferred and provide standardization through simple and rapid analysis^17^. Antibodies are essential tools for serological diagnosis and their use is growing rapidly^18,19^. However, conventional serological techniques cannot be used because of difficulty obtaining target-specific antigens; expression of target proteins may not occur, making purification impossible^20^. Paradoxically, animals or animal cell cultures are required for plant virus diagnosis in plant virus laboratories.

Recombinant antibodies have proven useful for diagnostics and research^19,21–23^. The most commonly used form of recombinant antibodies is the single-chain variable fragment (scFv) which has a simple structure and low molecular weight^24–26^. An scFv consists of a variable heavy chain (V_H_) and a light chain (V_L_) of the antibody and is connected by a short polypeptide linker^27^. It is easily displayed on a phage, and a library can generate appropriate new recombinant antibodies without purification and special equipment^28–30^. It is possible to select an scFv with superior and specific affinity for a target antigen through bio-panning^31,32^. In addition, an scFv can be easily expressed in *Escherichia coli*, which grows rapidly in a simple and inexpensive medium and can express significant amounts of a desired protein^23,33,34^. Yeast-surface displays have recently emerged as a powerful platform for protein engineering^35,36^. Yeast cells can not only efficiently express 50,000 copies, but also take advantage of unique eukaryotic post-translational machinery such as glycosylation and disulfide isomerization^37^. For this reason, yeast-surface displays were chosen to express antigens in this study. Based on a combination of yeast-surface and scFv-phage display^38^, we screened SPLCV-specific scFv clones by bio-panning. In this work, a novel detection system for the SPLCV geminivirus is proposed. The application of phage- and yeast-surface displays to the selection of virus-specific scFv antibodies to SPLCV is also discussed.

## Results

### Virus antigen detection and selection of yeast cells expressing viral antigen

**Figure 2:**
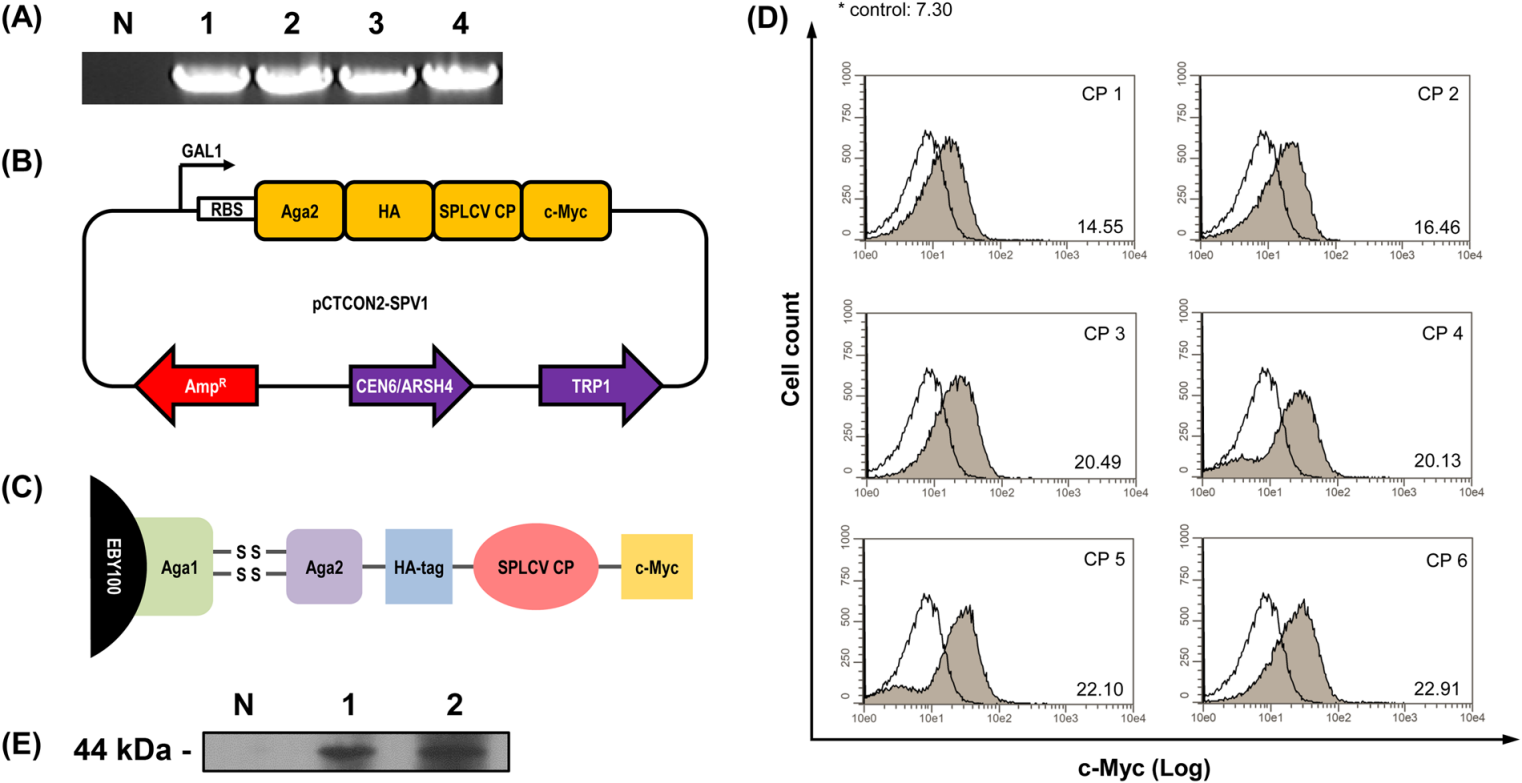
(**A**) PCR detection of target protein. The amplified sweet potato leaf curl virus (SPLCV) V1 gene was confirmed by 1% agarose gel electrophoresis. N lane is the no-template control and lanes 1–4 are amplified SPLCV V1 genes. **(B)** A schematic diagram of a plasmid (pCTCON-SPV1) for yeast-surface display used for antigen [SPLCV coat protein (CP)] expression. **(C)** A schematic diagram of a yeast-surface display. Antigen protein is expressed with yeast glycoprotein (Aga2) and multiple tags (HA, c-Myc). **(D)** FACS analysis of yeast cell lines displaying SPLCV V1. The x axis of each plot represents the cell count and the y-axis represents the intensity of the fluorescent signal of c-Myc-captured antibodies. The white graph is for the negative control cell, and gray histograms are for antigen-displaying induced cells. The x-mean values of antigen-displaying cells are shown at the bottom right of the plot. **(E)** Western blot analysis of the verification of antigen expression. The N lane is the negative control (EBY100), and lanes 1 and 2 are the induced samples (CP5, CP6). The anti-c-Myc antibody detected the tag fused to the antigen protein.

SPLCV is one of the monopartite begomoviruses in geminivirus family, and SPLCV has 6 ORFs for encoding different proteins for systemic movement (V1), cell-to-cell movement (V2), virus replication (C1), transcription activator (C2) or replication enhancer (C3), and symptom determinant (C4). V1 of SPLCV encodes coat protein which is the only structural protein of geminivirus particles. As a target antigen, V1 protein of SPLCV Haenam 1 strain was amplified by PCR and the products were visualized in the form of a slight single band product on 1% agarose gel containing ethidium bromide (Fig. 2A). The V1 sequence of SPLCV Haenam 1 strain consisted of 774 bp nucleotides. The amplified DNA fragments from the V1 of SPLCV was cloned into a pCTCON plasmid vector for yeast-surface display.

Yeast display has recently emerged as an alternative strategy, with one important advantage over phage display: the ability to precisely control selective parameters by FACS analysis. This technique can reliably quantify differences in antigen expression levels; antigens are fluorescently labeled with an antibody recognizing the C-terminal c-Myc tag encoded by the plasmid vector (Fig. 2B,C).

The surface display of SPLCV V1 by Saccharomyces cerevisiae also allows the detection of appropriately labeled antigen-antibody interactions by flow cytometry. The 6 colonies were evaluated by shifting degrees from wild-type yeast. The yeast expression cells grown in SDCAA media were used as controls. Representative flow cytometry histograms for selected clones are shown in Fig. 2D. The vertical axis indicates the cell number, and the horizontal axis indicates fluorescence. Similar Gaussian distribution patterns of all selected clones were observed. The mean value of the x axis (x-mean) was used to measure the degree of expression of SPLCV V1 as a statistic. Only one colony of SPLCV showed the shifting of the x-mean value from the control. The x-mean value of the control cell was 7.30 while the highest value of one colony was approximate three times higher (22.91). Two high-expression cell lines (CP 5 and CP 6) were analyzed by western blot to verify antigen expression (Fig. 2E). The anti-c-Myc antibody detected a C-terminal tag of target protein. The data showed a 44 kDa band corresponding to the expected size of SPLCV V1. No band was observed in EBY 100, which was used as the negative control.

### Screening of scFvs by bio-panning

**Figure 3:**
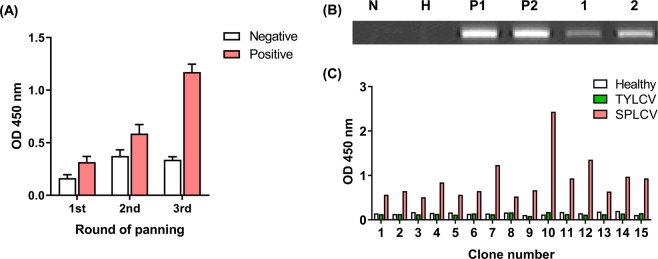
(**A**) The bio-panning result with sweet potato leaf curl virus (SPLCV) displayed on the yeast cells as an antigen. Sixty randomly selected colonies were measured after each panning round. The collected phages were bound to the target antigen and quantitatively confirmed using HRP-conjugated anti-M13 antibodies at an OD of 450 nm. Data are presented as means ± SEMs. **(B)** PCR detection of SPLCV in different tissues (phloem tissues and leaves) of sweet potatoes. Lane N is a no-template control. Lane H is virus-free sweet potato samples. Lane P1 and P2 are amplified with phloem tissue of SPLCV-infected samples, and 1, 2 are amplified viral DNA from sweet potato leaf samples. **(C)** Binding of scFv antibodies, determined by ELISA against SPLCV-infected plant leaves. SPLCV-infected sweet potato leaves were coated onto 96-well microtiter plates. Each scFv was detected using HRP-conjugated anti-M13 antibodies and TMB substrate solution. ELISA readings (OD_450_) were collected after 30 min of incubation in a TMB substrate at 25 °C. Clone numbers 10 and 12 are the selected scFv used for expression.

The antigen-binding affinity of randomly selected colonies was measured in each round to determine whether the affinity was higher by bio-panning rounds (Fig. 3A). OD_450_ was measured for screening of phage scFvs from randomly selected colonies. As the number of panning rounds increased, the binding affinity of positive libraries for positive antigens tended to increase. SPLCV-infected sweet potato leaves were used for the selection of antigen-specific scFv after bio-panning. SPLCV-infected sweet potato samples of leaves and phloem tissue^39^ were identified as shown in Fig. 3B. Both the sensitivity and the specificity used in the diagnosis were critical values. The negative samples (Healthy, TYLCV samples) had OD_450_ values of less than 0.20, and 15 phage scFv clones showed binding affinity with SPLCV- infected plants (Fig. 3C). The scFv DNA of selected clones was amplified by PCR and the nucleotide sequences were analyzed. The complementarity-determining regions (CDRs) were identified as shown in Table 1. The sequence was compared with the IgBLAST KABAT antibody sequence database^40^. Most of the scFv clones consisted of nonsense mutations or junk codons, and some clones could not be analyzed. A comparative analysis of VH and VL sequences showed significant differences in the CDRs, which are associated mainly with different biological activities^41^. Only two scFv clones (scFv “10” was named “G7”, and “12” was renamed “F7”) had complete sequences, including VH and VL chains.

**Table 1:** CDR sequences of selected scFv genes via IgBlast.

	V_H_			V_L_		
	CDR1	CDR2	CDR3	CDR1	CDR2	CDR3
4	TFYWT	YIDYSGTDYNPSLES	SYKAG	SGDKLGDKYAK	QDSKRPS	QAWDSST
7	TYEMN	YISSSGETMYYADSVKG	GKRAY	RTSQSVSNKLA	DVSTRAT	QQYNNWP
10	SYSIH	WINAASSNTRYSQKFED	TFGEMDGNFDY	RASQTISSTFLA	DASSRAT	*
12	DYAMT	TISGSGRYTYYADSVKG	DRVALAGTNYYGLDV	SGDKLPTKYVW	QDSIRPS	LGWDRST
14	TYEMN	YISSSGETMYYADSVKG	GAYKRG	TLRSGINVGTYRIY	YKSDSDKQQGS	MTWHSSA

### Bacterial expression and characterization of anti-SPLCV scFv

**Figure 4:**
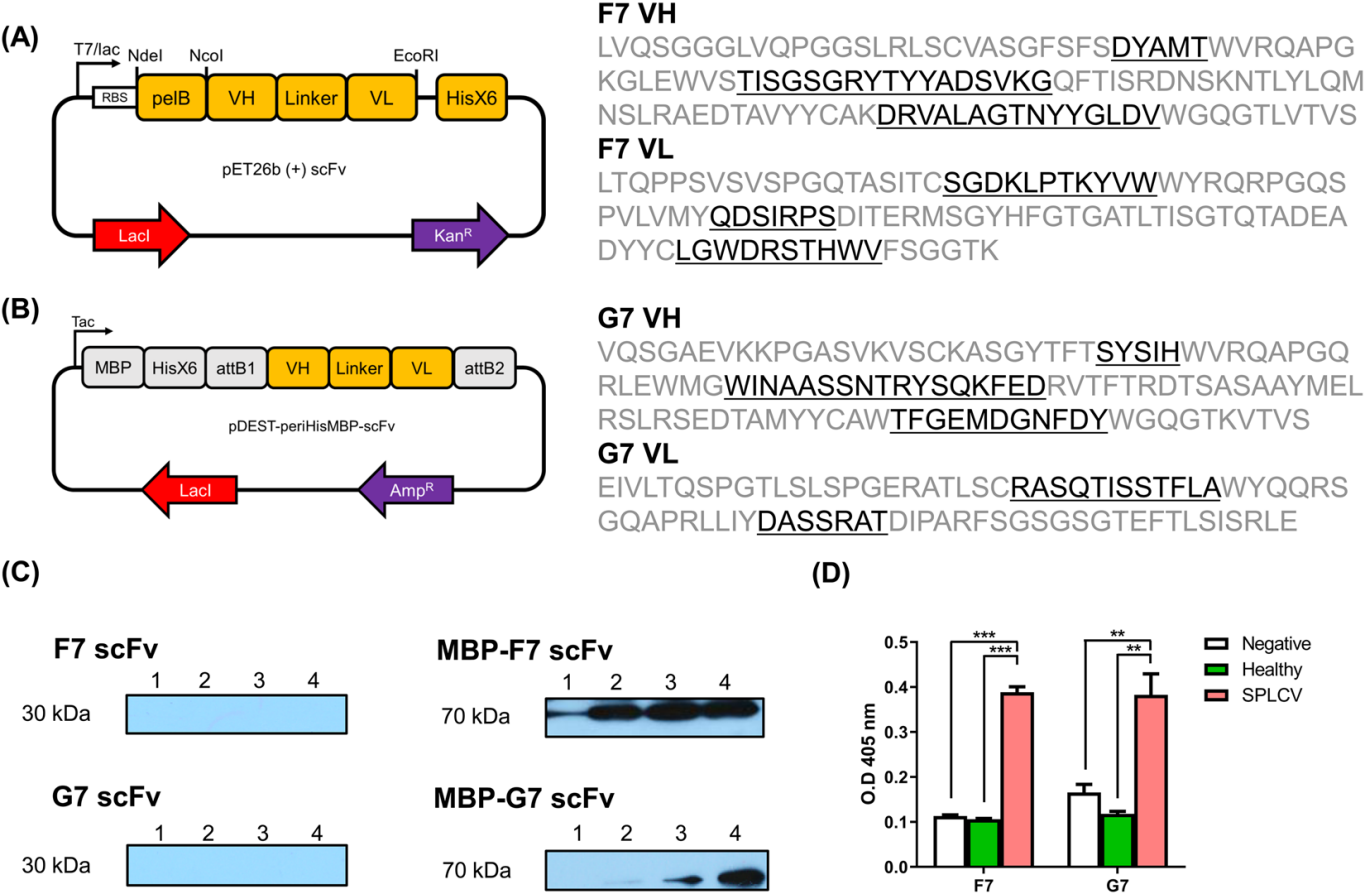
(**A**) Schematic diagram of pET26b (+) scFv plasmid vector for expression of scFv. **(B)** Schematic diagram of scFv and MBP fusion protein expression plasmids. **(C)** Expression tests of anti-sweet potato leaf curl virus (SPLCV) scFv with various IPTG concentrations. Lane 1 shows non-induction, lanes 2–4 show induction with IPTG (0.5, 1, and 2 mM). *E. coli* (BL21 [DE3] pLysE) cells were induced at OD_600_ = 0.6, 26 °C for 6 h. **(D)** Quantitative analysis of ELISA results using a spectrophotometer and data are presented as means ± SEMs (^**^P < 0.01, ^***^P < 0.001, GraphPad Prism, GraphPad).

The two scFv genes, F7 and G7, were subcloned into pET26b (+) (Fig. 4A) and pDEST-periHisMBP plasmids, respectively (Fig. 4B), and expressed in BL21 (DE3) pLysE. The expression test was performed under various IPTG concentrations (0, 0.1, 1, and 2 mM) and confirmed by western blot analysis (Fig. 4C). In the pET26b (+) plasmids, no soluble scFvs were expressed, and the plasmids were soluble in MBP fusion proteins. When the scFv protein was purified using Ni-NTA, the scFv did not bind well to the column. We therefore performed functional analysis using a filtrated medium in which scFv was expressed as soluble. To compare the binding affinity of different soluble scFv fragments, each protein was measured quantitatively by ELISA (Fig. 4D). Analysis of individual *P* values revealed a significant difference between negative and healthy samples (*P* < 0.0001, both) for F7 scFv. On the other hand, G7 scFv showed a slightly lower difference in the negative (*P* = 0.0049) and healthy (*P* = 0.0013) samples than did F7. Both scFv showed specific binding affinity to SPLCV sample.

### Avidity effects of bivalent scFv binding test

**Figure 5:**
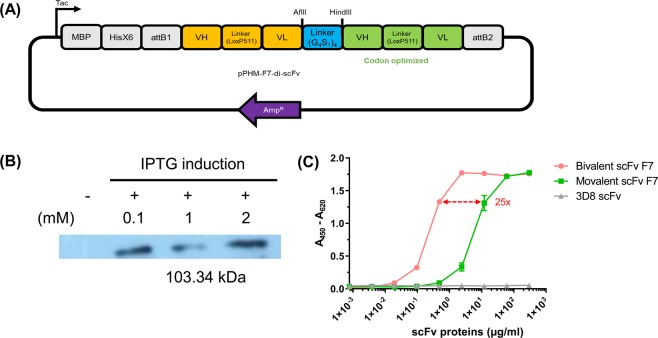
Expression test and avidity effects of bivalent scFv. **(A)** Plasmid vector map for bivalent scFv. This construct was designed based on a MBP fusion expression plasmid, pDEST-periHisMBP^64^. The existing F7 gene (shown in yellow) and the *E. coli* codon-optimized F7 gene (shown in green) were ligated with a glycine-serine linker (shown in blue). The *Afl*II and *Hin*dIII restriction enzyme sites that do not digest the backbone were added, so that the linker could be replaced. **(B)** Expression test of bivalent F7 scFv protein. The molecular weight of the expressed fusion proteins was 103.34 kDa in total and the molecular weight of each subunit was as follows: signal peptide (N-terminal signal peptide of MBP; 4.6 kDa) and MBP and polyhistidine tag (45.3 kDa). Lane 0 shows non-induction, lanes 1–3 show induction with IPTG (0.1, 1, and 2 mM). The induction was carried out at OD_600_ = 0.6, and overexpressed at 26 °C for 16 h. **(C)** Bivalent scFv (●) and monovalent scFv (■) were bonded with serial dilution in homogenized sweet potato leaf curl virus-infected leaf samples in coated ELISA wells. The 3D8 scFv (▲) was used as a negative control. Data are presented as means ± SEMs.

To increase antigen-binding affinity, bivalent F7 scFv was expressed and binding activity was confirmed. The bivalent scFv was expressed in BL21 (DE3) pLysE strain (Merck). The expression test was confirmed under various IPTG concentrations (0, 0.1, 1, and 1 mM). Expression of bivalent F7 scFv protein was confirmed by western blot (Fig. 5B).

Different scFv formats containing only F7 scFv domains including monovalent and bivalent scFv were compared in ELISA (Fig. 5C). The monovalent F7 protein bound only at relatively high concentrations of more than 2 μg/mL. The bivalent F7 scFv bound 25 times as efficiently to SPLCV samples compared with the monovalent scFv.

## Discussion

Generally, many of the conventional serological diagnosis tests by using polyclonal- or monoclonal antibodies are less specific and less sensitive than molecular diagnosis approaches. Therefore conventional serological diagnosis tests is not suitable for precise and accurate diagnosis of field samples but it is useful in serologically confirmatory testing and epidemiological studies^42^. To overcome the disadvantages associated with conventional serological diagnosis techniques, molecular diagnosis tests such as PCR, real-time PCR, recombinase polymerase amplification (RPA) and loop mediated isothermal amplification (LAMP) assays have been used for specific and sensitive detection of samples^43–45^. But still serological detection tests have many advantages to detect viruses easily and rapidly from many samples if suitable antibodies are provided. Recombinant scFv has been developed to overcome the limitations and drawbacks of conventional polyclonal- and/or monoclonal antibodies. In many review papers, recombinant scFv can provide us many advantages such as no animal immunization required, shorter periods for scFv production, much cheaper than conventional antibody for mass-production and easy maintaining the scFv as a gene in expression vectors^46^. Nowadays, scFv has a well-established protocol to produce a completely functional antigen-binding fragment in bacterial systems. In addition, the advances in scFv applications give us more efficient and generally applicable method to produce better scFv by an antibody engineering techniques.

To select an antigen-specific scFv with bio-panning, a large amount of antigen is required. We used yeast-surface display for this study because the system offers eukaryotic post-translational machinery such as disulfide isomerization and glycosylation^37^. In this study, we prepared a genetically engineered yeast cell that displays the SPLCV V1 as an antigen. Phage display has been used to develop target-specific recombinant antibodies^30,47^. Some advantages of phage display over conventional hybridoma techniques include shorter time, lower cost, and greater application^23^.

Based on a combination of yeast-surface display and scFv-phage display, we screened SPLCV-specific scFv clones by bio-panning as described in^48,49^. Two scFv clones were selected for SPLCV and these genes could be expressed in *E. coli* for mass production. The scFv was not expressed as soluble in *E.coli* with short fusion peptides such as a His tag, but was expressed in relatively large protein such as MBP. In addition, a small tag such as a His tag could be detected as an antibody in SDS-PAGE under denaturation conditions. However, the non-denatured protein was not purified through a Ni-NTA column. The His tag was likely not exposed to the outside due to the steric structure of the scFv proteins. The binding affinity for antigens and the properties of the expressed scFv clones were clearly identified by ELISA using SPLCV-infected plant leaves. The results showed that the expression of scFv in *E. coli* can induce reactivity and specificity of a recombinant antibody. Therefore SPLCV-specific scFv can be mass-produced easily and inexpensively in *E. coli*.

The design of a bivalent scFv-expressing vector enhanced the binding affinity of monovalent scFv protein^50,51^. The avidity effect of SPLCV-specific bivalent scFv-Fc proteins resulted in a 25-fold improvement in antigen binding compared with the monovalent scFv fragment. This recombinant scFv detection method with bivalent scFv will contribute to more efficient virus detection system development by enhancing scFv-antigen binding affinity. Then this method can detect virus from small piece of sweet potato samples compared the conventional serological virus detection method with monovalent scFv.

With these advantages, scFv could play an important role in SPLCV diagnosis. The main purpose of this study was screening specific antibodies for sweet potato viruses as a diagnostic method. Recombinant antibody fragments for specific antigens can be modified with genetic engineering to more specific and stable antibodies for serological methods of detection. In addition, a genetically engineered, recombinant scFv with higher efficiency can be produced, based on different scFv binding actions to the antigen.

## Methods

### Virus antigen DNA preparation

SPLCV Haenam 1 strain (GenBank No. HM754641)-infected sweet potato leaves (Fig. 1B) were homogenized in liquid nitrogen after sampling. The total genomic DNA of the sweet potato was extracted following a method described in^52^. A small amount of sweet potato (less than 1 g) was collected in a 1.5 mL microcentrifuge tube and 500 μL of Dellaporta extraction buffer (100 mM Tris, pH 8.0), 50 mM of ethylenediamine-tetraacetate EDTA, 500 mM of NaCl, ad 10 mM of β-mercaptoethanol (BME) were added. The microfuge tube was mixed vigorously and incubated for 10 min at 65 °C with 33 μL of 20% sodium dodecyl sulfate (SDS; w/v). Next, 160 μL of 5 M potassium acetate was added, mixed, and spun for 10 min at 15,000 *g* in a centrifuge. The 450 μL of supernatant was transferred to a new tube and the process was repeated until the supernatant was free of debris. Isopropanol (0.5 volumes) was added with a vortex and spun for 10 min at 15,000 *g*. When the supernatant was removed, all nucleic acids were in the bottom of the tube. The pellet was washed with 70% ethanol and spun repeatedly for 5 min at 9,000 *g*. After removal of the supernatant, the pellets were dried at room temperature for 30 min. Finally, the pellet was resuspended in 200 μL of RNase-treated TE buffer (10 mM Tris-HCl, 1 mM EDTA, pH 8.0). The extracted genomic DNA was purified and then stored at −86 °C.

### SPLCV coat protein gene amplification

SPLCV coat protein (V1) was amplified by PCR with genomic DNA as a template. The forward primer 5′-CCTAGGATGACAGGGCGAATTCCC-3′ and reverse primer 5′-AGATCTATTATTGTGCGAATCATAGAAA-3′ were designed to amplify the coat protein. The amplification reaction was performed in PCR tubes containing 1 μL of the template, 10 pmol of each primer and 2 × PCR premix (Takara, Tokyo, Japan), for a total volume of 20 μL. PCR amplification was performed with a thermal cycler machine (T100; BioRad, California, USA) under the following conditions: 10 min at 96 °C for pre-denaturation, followed by thermal cycling for 35 cycles (30 s at 96 °C, 30 s at 55 °C, and 1 min at 72 °C), 10 min at 72 °C for the final extension and storage at 12 °C. The amplified product of the V1 gene (774 bp) was identified on 1.5% agarose gel containing ethidium bromide. The DNA was purified with a gel extraction kit (Macrogen, Seoul, Republic of Korea). Purified DNA was cloned into the TA-cloning vector pGEM®-T easy (Promega, Madison, WI, USA) and introduced into *E. coli* DH5α according to the manufacturer’s instruction. After transformation, a single colony was placed onto a Luria-Bertani (LB) agar (1.5% w/v) plate containing 50 μg/mL of ampicillin, 100 μg/mL of X-gal, and 1 mM of isopropylthio-β-D-galactoside (IPTG). The selected colony was cultured in 3 mL of LB broth with 50 μg/mL of ampicillin, and the plasmid was extracted with a plasmid mini-prep kit (Bioneer, Daejeon, Republic of Korea) after cell incubation. The plasmid sequence was analyzed by the Macrogen-sequencing service (Seoul, Republic of Korea) with T7 and SP6 primers and a basic local alignment search tool (BLAST) from the National Center for Biotechnology Information (http://www.ncbi.nlm.nih.gov).

### Subcloning for yeast surface display

The SPLCV V1 fragment was inserted into the pCTCON plasmid. The V1 gene in T-vector was digested with *Avr*II and *Bgl*II (Takara, Tokyo, Japan), and inserted between the *Nhe*I to *Bam*HI sites of pCTCON according to the manufacturer’s instructions. The ligate was inserted into a DH5α-competent cell, and colony selection onto LB agar plate with ampicillin, colony culture selection in broth, mini-scale plasmid extraction, and sequencing analysis were performed.

### Yeast transformation and yeast surface display

Yeast (*Saccharomyces cerevisiae* strain EBY100)-competent cell preparation was performed following a Clontech manual (Tokyo, Japan)^53^. Pre-culture was carried out by streaking cells onto a yeast-peptone-glucose (YPD) plate containing 2% dextrose (w/v) and letting them grow for 3 days at 30 °C. A single colony was inoculated with fresh YPD and cultured to an optical density at 600 nm (OD_600_) of 1.5 and diluted 10-fold in YPD and grown at 30 °C to an OD_600_ of 0.4–0.6. The cells were harvested and washed with distilled water. The yeast was resuspended in sterilized TE/LiAc buffer (100 mM lithium acetate, 100 mM Tris-HCl, 10 mM EDTA, pH 7.5). The plasmid DNA containing V1 and cell were mixed with carrier DNA (sheared salmon sperm DNA) and sterilized PEG/LiAc (40% of PEG 4000, 100 mM lithium acetate, 100 mM Tris-HCl, 10 mM EDTA, pH 7.5) in a fresh tube with vortexing and incubated at 30 °C for 30 min with shaking at 200 rpm. Dimethyl sulfoxide was added and the solution was incubated for 15 min in a 42 °C water bath and left on ice for 1 min. Cells were then harvested at 15,000 *g* for 5 s and resuspended in a 1X TE buffer and separated in synthetic defined media containing a dropout supplement (without tryptophan). A single colony was grown in SDCAA media overnight. For the seed culture, the cell was spun by centrifuge at 2,500 *g* for 15 min, and the media changed to SGCAA with 2% galactose and cultivated for 3 days.

### Fluorescence-activated cell sorting analysis

SPLCV V1 protein-displaying yeast cells were analyzed by fluorescence-activated cell sorting (FACS) analysis. Cells (1.2 × 10^4^) were rinsed with phosphate-buffered saline (PBS; pH 7.4) containing 0.1% (w/v) of bovine serum albumin (BSA), and then blocked in PBS with 0.1% BSA (PBS-B). After blocking, the cells were treated in dried-milk PBS (PBS-M)–diluted monoclonal anti-c-Myc antibody (1:500, produced in mice; Sigma, St. Louis, MO, USA) for an hour at 25 °C and washed with PBS-B. The cells were then incubated in PBS-B–diluted polyclonal anti-mouse immunoglobulin G (IgG) TRITC-conjugated antibodies (1:500; Abcam, Cambridge, MA, USA) for 2 h at 4 °C. Cells were washed with PBS-B, and analyzed on a Guava EasyCyte mini instrument (Merck, Darmstadt, Germany) and data were analyzed using Cytosoft software (version 4.2.1). *Tomato yellow leaf curl virus* (TYLCV) V1displaying yeast cells, cultivated in SDCAA were used as controls.

### Yeast western blot analysis

Yeast media were spun and treated in a protein sample buffer (60 mM Tris-HCl, 25% glycerol, 2% SDS, 14.4 mM BME, 0.1% bromophenol blue, pH 6.8) for disulfide bond cleavage. Each supernatant was isolated by centrifuge and equal amounts were separated by 10% polyacrylamide gel electrophoresis (SDS-PAGE) and blotted onto a nitrocellulose membrane (GE healthcare, Chicago, IL, USA) with a Towbin buffer (25 mM Tris, 192 mM glycine, 0.1% SDS, 20% [v/v] methanol) with a semi-dry transfer unit. The membranes were rinsed with Tris-buffered saline (TBS; pH 7.4) containing 0.1% (v/v) Tween-20 as a washing buffer (TBS-T). The membranes were blocked with TBS-T with 3% (w/v) skim milk (blocking buffer) and reacted with a blocking buffer-diluted anti-c-Myc monoclonal antibody (1:2000; Sigma, St. Louis, MO, USA) and anti-mouse IgG horseradish peroxidase (HRP)-conjugated antibodies (1:2000; Cell Signaling Technology, Danvers, MA, USA). The membrane-developing reaction was performed with enhanced chemiluminescence solution (GenDEPOT, Katy, TX, USA).

### Bio-panning with phage-displayed scFv libraries

A higher initial antibody library size significantly increases the selectivity of high-affinity antigen-specific antibodies^54–56^. The human scFv library pRGA-scFv was provided by Prof. Myung-Hee Kwon (Department of Microbiology, Ajou University School of Medicine)^57^. The scFv gene fragments were inserted between the *Sfi* I and *Not* I restriction sites of a modified pCANTAB 5E phagemid vector. Titers of the propagated phage library were measured as kanamycin-resistant colony forming units (CFUs). Selection of anti-SPLCV phage scFvs by bio-panning was performed as described in^48,49^. Briefly, wells of 96-well plate (SPL Life Sciences, Pocheon, Korea) were coated with the yeast cells [blank (PBS), negative samples (TYLCV V1–displaying yeast cells) and positive samples (SPLCV V1–displaying yeast cells)] diluted to OD_600_ value of 0.6 with carbonate coating buffer (pH 9.6). After that, cover the plate with wet paper towel and incubate at 4 °C overnight. After a brief washing with TBS-T buffer (0.1% Tween 20 in TBS), all wells were blocked with blocking buffer (TBS, 0.1% Tween 20, 3% skim milk) for 2 h at 37 °C. For selection of phage scFv proteins, 10^8^ mL/CFU recombinant phages in blocking buffer were added in blank wells, and incubated for 2 h at room temperature. Non-binding phages were transferred to negative-sample wells and incubated overnight at 4 °C. Collected non-binding phages were transferred to positive-sample wells and incubated for 1 h at room temperature. The positive-sample wells were washed vigorously with TBS-T 6 times. SPLCV-binding recombinant phages were eluted with 100 mM triethylamine and neutralized with 1 M Tris-HCl (pH 7.0). The eluted phages was titrated with XL1-blue (Agilent, Santa Clara, CA, USA) CFUs on an LB agar plate containing tetracycline (50 μg/mL) and carbenicillin (50 μg/mL). Three rounds of bio-panning were carried out, and phage titration was performed at every panning round.

### Isolation and production of SPLCV-specific scFv

Randomly selected XL1-blue colonies containing individual clones from each bio-panning round were grown in 96-well mini tubes for 6 h at 37 °C and shaken with 2TY growth media containing 25 μg/mL tetracycline, 50 μg/mL carbenicillin, and 1% (w/v) glucose. After incubation, the cells were inoculated into new tubes with fresh 2TY growth media and incubated for 1 h at 37 °C with shaking. The M13KO7 helper phage was inoculated to cells at a multiplicity of infection of 50 and incubated 1 h at 37 °C without shaking. The soup was removed after centrifuging at 2,500 *g*, and resuspended in 2TY broth containing 25 μg/mL tetracycline, 50 μg/mL carbenicillin and 50 μg/mL kanamycin overnight at 30 °C.

### Phage ELISA

To select putative antigen-binding phage scFv clones, a phage enzyme-linked immunosorbent assay (ELISA) assay was performed in 96-well microplates coated with sweet potato leaves (and healthy leaves as controls) in a general-extraction buffer (10 mM sodium sulfite, 2% (w/v) polyvinylpyrrolidone (MW 40,000), 0.2% (w/v) sodium azide, 2% (w/v) BSA, 2% (v/v) tween-20 with PBS, pH 7.4; GEB)^58^ overnight at 4 °C. Following rinsing with TBS-T 6 times, each phage clone culture’s supernatants were added to each antigen and control well and incubated for 2 h at room temperature. The wells were washed 3 times with TBS-T, and the plates were incubated with HRP-conjugated anti-M13 (1:1000; 11793, Sino Biological Inc., Beijing, China) antibodies at room temperature for 1 h. After washing 6 times with TBS-T, TMB substrate solution (Agdia) was added for 30 min. The enzymatic reaction was stopped by the addition of 1 M sulfuric acid. Absorbance was read at OD_450_ using a microplate spectrophotometer (BioTeK, Winooski, VT, USA).

### Sequence analysis of selected SPLCV-specific phage scFv

Selected scFv clones by bio-panning were re-grown in 2TY media containing tetracycline and carbenicillin, and plasmid extraction was performed. Using these plasmids as a template, scFv sequences were amplified by PCR under the following conditions: 5 min at 96 °C for denaturation, followed by thermal cycling for 35 cycles (30 s at 96 °C, 1 min and 30 s at 55 °C and 1 min at 72 °C), and 5 min at 72 °C for the final extension. PCR was performed by 2X premix (Takara, Tokyo, Japan) using the primer sets pCANTAB5E-S1 5′-CAACGTGAAAAAATTATTATTCGC-3′ and pCANTAB5E-S6 5′-CATTTACTTAAAAGACATACTCC-3′. The amplified DNA was sent to Macrogen (Seoul, Republic of Korea) and analyzed with a DNA-standard sequencing service. Amino acid sequences were deduced from the obtained nucleotide data (ExPASy, https://web.expasy.org/translate). From the results of sequence analysis of screened scFv bound to each antigen, complementarity-determining regions (CDRs) of scFv were found with IgBlast (https://www.ncbi.nlm.nih.gov/igblast)^40,59^.

### Bacterial expression of scFv

The selected scFv genes were cloned into a pET26b (+) (Merck) vector and expressed in *E. coli*. The genes of scFv were amplified by PCR using primers (Table 2) that added *Nco*I and *Eco*RI sites for the pET26b (+) plasmid to the 5′ and 3′ ends of the PCR product, respectively. The His-tag for pET26b (+) at the C-terminus was used for purification. The digested gene products were separated on 1% (w/v) agarose gels and the DNA was excised using a gel extraction kit (Bioneer). The digested PCR products and vectors were ligated overnight at 16 °C and inserted into *E. coli* strain BL21 (DE3) pLysE cells (Merck). To optimize the expression condition, 3 mL of exponential growth culture (with an OD_600_ of approximately 0.6) was induced at 26 °C overnight with 0.1, 1, and 2 mM IPTG in a shaking incubator. Large-scale expression of scFv genes in 200 mL of LB medium was induced by the addition of IPTG during 6 h of growth at 26 °C. The cells were harvested by centrifugation (2,500 *g*, 4 °C for 15 min) and the proteins were extracted from cell periplasm by cold osmotic shock^60^ and filtrated with a vacuum filter (Merck). For purification, supernatants of scFv expressed in pET26b(+) plasmids were applied to an nickel-nitrilotriacetic acid (Ni-NTA)^61^. 10% SDS-PAGE was performed to identify and separate each protein. Western blot analysis was carried out with primary anti-His–tag mouse monoclonal antibodies (1:5,000; R&D Systems, Minneapolis, MN, USA) and HRP-conjugated secondary anti-mouse IgG (Cell Signaling Technology) to detect scFv proteins.

**Table 2:** Nucleotide sequence of the primer sets used for cloning of scFvs.

Primer name	Sequences (5′-3′)
SPLCV_F7_scFv_26b_F	CCA TGG ATC TGG TGC AGT CTG GGG
SPLCV_F7_scFv_26b_R	GAA TTC GGG GTG ACC TTG GTC CCT C
SPLCV_G7_scFv_26b_F	CCA TGG ATG TGC AGT CTG GGG CTG A
SPLCV_G7_scFv_26b_R	GAA TTC GGG GCT CCA GTC TGC TGA T

### Maltose-binding protein fusion expression

To improve protein expression, the scFv genes were subcloned into maltose-binding protein (MBP) encoding a plasmid vector, pDEST-periHisMBP (Addgene, 11086, Cambridge, MA, USA)^62^ using gateway cloning^63^. The scFv genes were amplified by PCR using primers (Table 3) in which the *AttB*1 and *AttB*2 sites were added to the 5′ and 3′ ends of the PCR product, respectively, for recombination site insertion. To produce entry clones, the BP reaction was carried out with BP Clonase™ II Enzyme mix (Invitrogen, Carlsbad, CA, USA) and pDONR221 (Invitrogen) as a donor plasmid. After addition of proteinase K, recombinant plasmids were transformed with DH5α and selected on the LB media containing kanamycin (50 μg/mL). Subcloning of an entry clone into a destination vector, pDEST-periHisMBP, was performed by LR Clonase™ II Enzyme mix (Invitrogen). After addition of proteinase K, the expression clones were transformed with DH5α and selected in an ampicillin (50 μg/mL) selection medium. The expression clone sequences were analyzed using a DNA sequencing service (Macrogen, Seoul, Republic of Korea). MBP-fusion scFvs were expressed in BL21 (DE3) pLysE cells (Merck). An expression test was then carried out as with the pET26b (+) clone.

**Table 3:** Nucleotide sequence of the primer sets used for MBP fusion expression.

Primer name	Sequences (5′-3′)
SPLCV_F7_attB1	GGG GAC AAG TTT GTA CAA AAA AGC AGG CTT CCT GGT GCA GTC TGG GGG AGG
SPLCV_F7_attB2	GGG GAC CAC TTT GTA CAA GAA AGC TGG GTC CTA GGT GAC CTT GGT CCC TCC GC
SPLCV_G7_attB1	GGG GAC AAG TTT GTA CAA AAA AGC AGG CTT CGT GCA GTC TGG GGC TGA GGT
SPLCV_G7_attB2	GGG GAC CAC TTT GTA CAA GAA AGC TGG GTC CTA GGC TCC AGT CTG CTG ATG GA

### Functional analysis of scFv proteins

To determine the binding activity of scFvs purified from *E.coli*, indirect ELISA was carried out. Briefly, sweet potato samples were coated on a 96-well microplate directly with a general extract buffer overnight at 4 °C. Following washing with TBS-T 6 times, the microplate was blocked with TBS-T containing 3% BSA solution and incubated for 2 h at room temperature. The wells were washed 3 times with TBS-T. The microplates were then coated with purified scFv proteins diluted in a blocking buffer at a concentration of 0.1 μg/μL for 1 h at room temperature. Following rinsing with TBS-T, anti-His tag mouse monoclonal antibodies (R&D Systems) and HRP-conjugated secondary anti-mouse IgG (Cell Signaling Technology) were applied to detect scFv proteins. After washing 6 times with TBS-T, TMB substrate solution (Agdia) was added for 30 min, and the enzymatic action was stopped with the addition of 1 M sulfuric acid. The absorbance was read at OD_405_ using a microplate spectrophotometer (Tecan Sunrise, Tecan, Switzerland).

### Cloning and soluble expression of homodimer scFv

The synthesized gene was cloned into maltose-binding protein (MBP), encoding a plasmid vector, pDEST-periHisMBP (Addgene, 11086, Cambridge, MA, USA)^62^, by gateway cloning^63^. The BP reaction was carried out with BP Clonase™ II Enzyme mix (Invitrogen, Carlsbad, CA, USA) and pDONR221 (Invitrogen) as a donor plasmid. The proteinase K was added and, recombinant plasmids were transformed into DH5α and selected on the LB agar plates containing kanamycin (50 μg/mL). To produce expression clones, additional recombination was performed by LR Clonase™ II Enzyme mix (Invitrogen) with entry clones and pDEST-periHisMBP. After addition of proteinase K, the expression clones were transformed into DH5α and selected on LB agar plates containing ampicillin (50 μg/mL) selection medium. The expression clone, named pPHM-F7-di-scFv (Fig. 5A), was analyzed by a DNA sequencing service (Macrogen, Seoul, Republic of Korea). Bivalent F7 scFv protein was expressed in BL21 (DE3) pLysE cells (Merck). An expression test was then carried out as for the protein A fusion clone. For purification, the supernatant products of scFv expression were applied to a nickel-nitrilotriacetic (Ni-NTA) agarose column (Thermo Scientific, Waltham, MA, USA)^64^. SDS-PAGE (10%) was performed to identify and separate proteins. Western blot analysis was conducted as described by^65^ using primary anti-His tag mouse monoclonal antibodies (1:5000; R&D systems, Minneapolis, MN, USA) and HRP-conjugated secondary anti-mouse IgG (Cell Signaling Technology) to detect target proteins.

### Functional analysis of bivalent scFv

To determine the binding activity of bivalent F7 scFv, indirect ELISA was carried out. Sweet potato samples were coated on a 96-well microplate directly with GEB at 4 °C for overnight in a humidified container. After washing six times with TBS-T, the microplate was blocked with TBS-T containing 3% BSA solution and incubated at room temperature for 2 h. The wells were rinsed three times with TBS-T. The microplates were then coated with purified bivalent scFv serially diluted in blocking buffer (five-fold dilution from 300 μg/mL) at room temperature for 1 hour. The serially diluted F7 scFv also used to compare. Each concentration of scFv protein was determined after calculation based on the values measured with a spectrophotometer with the molecular weight of the scFv protein and coefficient factor. We used estimates based on the values measured with a spectrophotometer to determine the protein concentration

Following rinsing with TBS-T, anti-His tag mouse monoclonal antibodies (1:1000; R&D systems) and HRP-conjugated anti-mouse IgG antibodies (1:1000; Cell Signaling Technology) were treated for detection. After six more rounds of washing, TMB substrate solution (Agdia, Evry, France) was added for 30 min, and the action was stopped by the addition of 1 M sulfuric acid. Absorbance was read at 405 nm against a reference wavelength of 620 nm (A450-A620) using a microplate spectrophotometer.
